# Impacts of long‐term elevated atmospheric CO_2_ concentrations on communities of arbuscular mycorrhizal fungi

**DOI:** 10.1111/mec.15160

**Published:** 2019-07-17

**Authors:** Irena Maček, Dave R. Clark, Nataša Šibanc, Gerald Moser, Dominik Vodnik, Christoph Müller, Alex J. Dumbrell

**Affiliations:** ^1^ Biotechnical Faculty University of Ljubljana Ljubljana Slovenia; ^2^ Faculty of Mathematics, Natural Sciences and Information Technologies (FAMNIT) University of Primorska Koper Slovenia; ^3^ School of Biological Sciences University of Essex Colchester UK; ^4^ Slovenian Forestry Institute Ljubljana Slovenia; ^5^ Department of Plant Ecology Justus‐Liebig University Giessen Giessen Germany; ^6^ School of Biology and Environmental Science and Earth Institute University College Dublin Dublin Ireland

**Keywords:** biodiversity, climate change, elevated CO_2_, long‐term experiments, microbial diversity, next‐generation sequencing

## Abstract

The ecological impacts of long‐term elevated atmospheric CO_2_ (eCO_2_) levels on soil microbiota remain largely unknown. This is particularly true for the arbuscular mycorrhizal (AM) fungi, which form mutualistic associations with over two‐thirds of terrestrial plant species and are entirely dependent on their plant hosts for carbon. Here, we use high‐resolution amplicon sequencing (Illumina, HiSeq) to quantify the response of AM fungal communities to the longest running (>15 years) free‐air carbon dioxide enrichment (FACE) experiment in the Northern Hemisphere (GiFACE); providing the first evaluation of these responses from old‐growth (>100 years) semi‐natural grasslands subjected to a 20% increase in atmospheric CO_2_. eCO_2_ significantly increased AM fungal richness but had a less‐pronounced impact on the composition of their communities. However, while broader changes in community composition were not observed, more subtle responses of specific AM fungal taxa were with populations both increasing and decreasing in abundance in response to eCO_2_. Most population‐level responses to eCO_2_ were not consistent through time, with a significant interaction between sampling time and eCO_2_ treatment being observed. This suggests that the temporal dynamics of AM fungal populations may be disturbed by anthropogenic stressors. As AM fungi are functionally differentiated, with different taxa providing different benefits to host plants, changes in population densities in response to eCO_2_ may significantly impact terrestrial plant communities and their productivity. Thus, predictions regarding future terrestrial ecosystems must consider changes both aboveground and belowground, but avoid relying on broad‐scale community‐level responses of soil microbes observed on single occasions.

## 1 INTRODUCTION

Global atmospheric CO_2_ concentrations have steadily increased from 280 to over 400 ppm since the middle of the 19th century, contributing to elevated global temperatures and other climate change effects (Donat, Lowry, Alexander, O'Gorman, & Maher, 2016; IPCC, 2014). Atmospheric CO_2_ concentrations will continue to rise and likely reach 750 ppm by 2100 (IPCC, 2014), leading to further environmental changes. However, the ecological impacts of elevated CO_2_ (eCO_2_) remain poorly studied across many taxa and ecosystems. While studying the response to eCO_2_ of the aboveground component of terrestrial systems has provided significant insights into many plant physiological processes (Becklin, Walker, Way, & Ward, 2017; Obermeier et al., 2017), the effects on belowground communities and their interactions remain largely unknown (Becklin et al., 2017; Blankinship, Niklaus, & Hungate, 2011; van der Putten et al., 2016). Yet, increases in photosynthetic activity under eCO_2_ (Ainsworth & Rogers, 2007) will increase photosynthate transfer to plant roots and the flow of carbon through soil microbial communities (Cheng et al., 2012; Drigo et al., 2010, 2013; Staddon et al., 2014), potentially altering their composition and dynamics (Johnson, Angelard, Sanders, & Kiers, 2013).

One functionally important group of soil microbes, with communities likely to respond to changes in photosynthate supply belowground, is the arbuscular mycorrhizal (AM) fungi (Drigo et al., 2010, 2013; Dumbrell, Nelson, Helgason, Dytham, & Fitter, 2010a). AM fungi are obligately symbiotic endobiotrophs of approximately two‐thirds of all land plants (Fitter & Moyersoen, 1996) and are entirely dependent on plant photosynthates for their carbon source (Smith & Read, 2008). As AM fungi inhabit plant roots, they receive increased levels of photosynthates under eCO_2_ before other soil microbes (Drigo et al., 2010, 2013) and previous research has suggested this leads to increased arbuscular mycorrhizal fungal colonization under eCO_2_ (Alberton, Kuyper, & Gorissen, 2005; Staddon, Jakobsen, & Blum, 2004; Treseder, 2004). Higher levels of fungal root colonization alongside the presence of arbuscules indicate active symbiosis and physiological compatibility between symbionts, and thus, increases in plant carbon supply to the AM fungi can trigger increased reciprocation in phosphorus and nitrogen to the plant, strengthening and stabilizing the mutualism (Fellbaum et al., 2012, 2014). However, how these eCO_2_ effects translate into changes in the diversity and composition of AM fungal communities in natural ecosystems is unclear (Veresoglou, Anderson, de Sousa, Hempel, & Rillig, 2016).

Morphological traits of mycorrhizal fungal root structures are not sufficient for taxa identification (Merryweather & Fitter, 1998); thus, for studies of AM fungal populations and communities, molecular methods have routinely been used since their inception (Helgason, Daniell, Husband, Fitter, & Young, 1998). However, these methods have yet to be applied to a long‐term experiment within a natural (as opposed to agricultural) system that examines the ecological impacts of eCO_2_. Within an agricultural soy monoculture free‐air carbon dioxide enrichment (FACE) system, Cotton, Fitter, Miller, Dumbrell, and Helgason (2015) used molecular methods and revealed increases in abundance of fast‐growing r‐strategists from the Glomeraceae family (Boddington & Dodd, 1999), which likely benefit most from increased availability of rhizosphere carbon. In contrast, slower growing K‐strategists (Boddington & Dodd, 1999; de Souza, Dalpé, Declerck, de la Providencia, & Séjalon‐Delmas, 2005) decrease in abundance within the same system. Previous research has suggested increased availability of photosynthetic carbon may decrease the evenness of AM fungal communities, as release from resource limitation often drives competitive exclusion (Dumbrell et al., 2011, 2010a). However, despite significant increases in specific AM fungal taxa in agricultural soy monoculture grown under eCO_2_, changes in community evenness were not observed and were probably suppressed by crop rotational practices (Cotton et al., 2015). This is not necessarily unexpected, as inconsistent responses of fungal diversity, including in a few cases AM fungi, to eCO_2_ are also reported across the only ten studies to examine this (Veresoglou et al., 2016); for recent review, see Cotton (2018). However, in these studies AM fungal responses to eCO_2_ were tested at the community level, ignoring the subtler population‐level dynamics throughout the plant growth period that may be affected by eCO_2_. In addition, community and population ecology data from long‐term CO_2_ fumigation experiments are largely missing (Veresoglou et al., 2016), and thus, established longer‐term effects may have been missed.

In this study, we investigate the impact of long‐term exposure (>15 years) to eCO_2_ on the indigenous communities of AM fungi in the semi‐natural grassland ecosystems of the GiFACE experiment. Using next‐generation sequencing (NGS; Illumina HiSeq), we quantified the response of AM fungal communities to eCO_2_ and tested the following hypotheses: (a) colonization of plant roots by AM fungi will increase under eCO_2_, reflecting stronger mycorrhizal mutualisms; (b) AM fungal responses to eCO_2_ will be taxon dependent, with increases in population densities of fast‐growing Glomeraceae (r‐strategists) relative to slower growing groups of AM fungi (K‐strategists; e.g., Gigasporaceae); and (c) while subtle changes in population densities are likely to be observed in response to eCO_2_, complete species turnover of AM fungal communities will not, but significant increases in the abundances of specific AM fungal taxa will reduce the evenness of their communities.

## 2 MATERIALS AND METHODS

### 2.1 Giessen free‐air carbon dioxide enrichment experiment

Initiated in 1998, the Giessen free‐air carbon dioxide enrichment (GiFACE) study – located in a semi‐natural, nongrazed grassland near Giessen (50°32′N; 8°41.3′E; 172 m above sea level), Germany (Jäger et al., 2003; Obermeier et al., 2017) – is known to be the longest running FACE experiment on a grassland ecosystem in the Northern Hemisphere (Jäger et al., 2003). The long‐term effects of eCO_2_ (+20% of ambient CO_2_) have been investigated in the GiFACE and have shown an enhanced aboveground production of plant biomass (Obermeier et al., 2017) and strong positive feedback effects on traits such as ecosystem respiration (Keidel, Kammann, Grünhage, Moser, & Müller, 2015) and nitrous oxide (N_2_O) production (Kammann, Müller, Grünhage, & Jäger, 2008; Regan et al., 2011). The GiFACE experiment is one of the rare experiments set in an old‐growth and predominantly C_3_ perennial‐dominated grassland that has been kept largely undisturbed for over 100 years. Importantly, the dominance of plant species and the composition of plant communities at GiFACE do not differ between eCO_2_ and aCO_2_ FACE rings (Kammann, Grünhage, Grüters, Janze, & Jäger, 2005; Obermeier, Lehnert, Ivanov, Luterbacher, & Bendix, 2018), controlling for effects on AM fungal communities that could be driven by changes in host species.

The study site receives ca. 644 mm annual mean precipitation with a mean annual temperature of 9.9°C (Abbasi & Müller, 2011). The permanent grassland has not been ploughed during the last 100 years, and since 1995, the experiment has been managed with fertilizer (40 kg N ha^−1^ annum^−1^ from calcium ammonium nitrate, reducing potential nutrient limitations). Within the FACE rings, the grass was manually cut twice per year with garden scissors (3–5 cm above the ground; Andresen et al., 2018). The grassland is an *Arrhenatheretum elatioris* Br.Bl. *Filipendula ulmaria* subcommunity, dominated by 12 grass species, two legumes and 15 nonleguminous herbs (Abbasi & Müller, 2011; see Table S1 for a full species list of the predominately perennial plant species present in the GiFACE rings in May 2013). The soil is classified as a Fluvic Gleysol and has a sandy clay loam texture over a clay layer, containing on average 4.5% C and 0.45% N (Abbasi & Müller, 2011; Müller et al., 2009; Table S2). The GiFACE experiment comprises 6 FACE rings with an inner diameter of 8 m, arranged in three paired blocks containing an eCO_2_ and an ambient CO_2_ (aCO_2_) FACE ring (Figure S1). eCO_2_ FACE rings maintain CO_2_ concentrations at ca. 20% above ambient through fumigation with CO_2_ for 1–13 hr daily depending on the month and prevailing wind directions (Tables S3 and S4).

### 2.2 Plant root sampling and AM fungal root colonization

Soil cores (84 cores, diameter ca. 100 mm; depth ca. 150 mm) were collected from three aCO_2_ (A1, A2 and A3) and three eCO_2_ rings (E1, E2 and E3). Soil core sampling was designed to cover both spatial and temporal variation in AM fungal communities. Eight soil cores were collected along the full length of the inner circle from each ring on 8 May 2013 (*spatial sampling*), with two additional soil cores collected from each ring on 7 May, 4 July and 30 September 2013 (*temporal sampling*). Mixed plant roots were extracted from each soil core and washed. Only living roots were used for further analyses (visual inspection). A random subsample of ca. 60% of roots was dried at 40°C and stored at room temperature prior to downstream DNA processing. Remaining roots were stored in 70% ethanol to quantify AM fungal root colonization. To assess AM fungal colonization, roots were cleared with hot 10% KOH and acidified with 1 N HCl. The intraradical fungal tissue was stained with 0.05% trypan blue in lactoglycerol. A detailed estimation of AM fungal structures (arbuscules and intraradical hyphae) in the stained roots followed Trouvelot, Kough, and Gianinazzi‐Pearson (1986) and used an Olympus Provis AX70 microscope (200× total magnification, *n* = 30 mixed 1 cm long root segments from each sampled core).

### 2.3 Molecular methods

In order to quantify the AM fungal community, mixed plant roots were homogenized using a Retsch mixer mill (Retsch). DNA was extracted from a 50 mg dry subsample of the homogenized roots using MoBio PowerPlant DNA isolation kits (MoBio Laboratories, Inc.), following the manufacturer's instructions. AM fungal communities were quantified using Illumina HiSeq NGS of amplicons of the SSU (small subunit) rRNA gene, which is a frequently used marker gene in studies of AM fungal diversity (Cotton, Dumbrell, & Helgason, 2014; Davison et al., 2015; Dumbrell et al., 2010a; Maček et al., 2011).

To produce amplicon libraries for Illumina HiSeq NGS, a 550‐bp fragment of the SSU rRNA gene was first amplified by PCR using Kapa HiFi Hot Start ReadyMix PCR Kit (KAPA Biosystems), the universal eukaryotic primer NS31 (Simon, Lalonde, & Bruns, 1992) and the primer AM1, which excludes plants, amplifies the major AM fungal families (Helgason et al., 1998) and provides accurate repeatability with no detectable PCR biases (Cotton et al., 2014). Forward and reverse primers were modified to contain Illumina‐specific overhang adapter sequences (Sigma). PCR was carried out in a 25 µl reaction volume with 2.5 µl of DNA template, 12.5 µl of Ready Mix, 3 µM of each primer (PCR conditions: 95°C for 3 min; 32 cycles at 95°C for 45 s, 62°C for 45 s and 72°C for 1 min; and 72°C for 5 min) on a Applied Biosystems Veriti Thermal Cycler (Thermo Fisher Scientific). PCR products were purified using Agencourt AMPure XP magnetic beads (Beckman Coulter). A secondary indexing PCR was then used to attach Illumina sequencing adapters and multiplex indexes, using the Nextera XT Index Kit (Illumina) and following Illumina's recommended protocols. Secondary PCR products were purified using Agencourt AMPure XP magnetic beads (Beckman Coulter), before quantification using a PicoGreen Assay on a NanoDrop 3300 Fluorospectrometer (Thermo Scientific). Equimolar concentrations of 75 successfully amplified samples were pooled and sequenced on an Illumina HiSeq 2500 running in rapid run mode with V3 2 × 300 bp paired‐end chemistry at The Earlham Institute, UK (formerly The Genome Analysis Centre).

### 2.4 Bioinformatic analyses

HiSeq reads were analysed following full guidelines for paired‐read Illumina amplicon libraries in Dumbrell, Ferguson, and Clark (2016). Briefly, quality control of NGS data was performed following recommendations described in Schirmer et al. (2015). Raw reads were first quality trimmed using sickle version 1.33 (Joshi & Fass, 2011), using the default Q20 quality threshold in paired‐end mode, discarding reads with ambiguous bases (Ns), and trimming only from the 3′ end. Quality trimmed reads were then subjected to error correction using BayesHammer (Nikolenko, Korobeynikov, & Alekseyev, 2013) implemented with default settings in SPAdes v3.7.1 (Nurk et al., 2013). Forward and reverse reads were then paired‐end aligned and primers removed using the PEAR algorithm (Zhang, Kobert, Flouri, & Stamatakis, 2014) implemented in PANDAseq version 1.33 (Masella, Bartram, Truszkowski, Brown, & Neufeld, 2012). To remove overly short or poorly aligned reads, we imposed a length filter; this removed reads that were shorter than 95% of the target amplicon's length, using custom Linux shell commands. Paired reads were then de‐replicated, sorted by abundance and clustered into operational taxonomic units (OTUs) at a 97% similarity threshold, and chimeras were removed using vsearch v2.1.2 (Rognes, Flouri, Nichols, Quince, & Mahé, 2016). Low abundance OTUs (<3 occurrences) were also removed as these are more likely to be nonbiological (Flynn, Brown, Chain, MacIsaac, & Cristescu, 2015). All aforementioned analyses were conducted using the bio‐linux 8 operating system (Field et al., 2006).

Representative sequences from each OTU were compared to the MaarjAM database (Öpik et al., 2010) using blast (Altschul, Gish, Miller, Myers, & Lipman, 1990), in order to determine their closest matched virtual taxon (VT; Öpik et al., 2010), and to identify non‐AM fungal OTUs, which were subsequently removed from further analyses. The representative sequence of the closest matched VT to each OTU was extracted from the MaarjAM database, alongside the closest matched sequences to each OTU identified from the NCBI database; these sequences were then aligned using ClustalW (Thompson, Gibson, Plewniak, Jeanmougin, & Higgins, 1997) and a neighbour‐joining phylogeny (Saitou & Nei, 1987), based on a Jukes–Cantor (Jukes & Cantor, 1969) substitution model and with *Geosiphon pyriformis* (Gehrig, Schüßler, & Kluge, 1996) as a specific outgroup to the AM fungi as well as *Corallochytrium limacisporum*, a choanozoan, as a general outgroup to all fungi (Vandenkoornhuyse, Baldauf, Leyval, Straczek, & Young, 2002), was constructed. Phylogenetic support was calculated via bootstrapping with 10,000 pseudo‐replicates (Felsenstein, 1985). All phylogenetic analyses were performed using geneious version 5.5.7 (Kearse et al., 2012).

### 2.5 Data analysis

**Figure 1:**
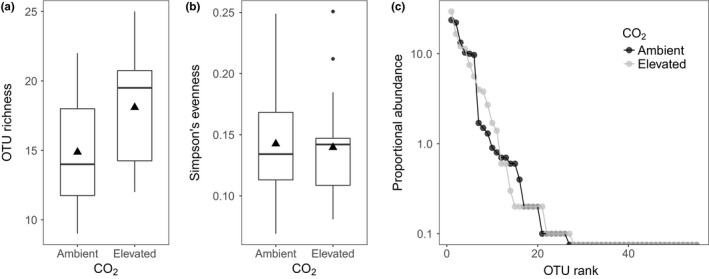
Differences in (a) AM fungal OTU richness, (b) Simpson's community evenness and (c) rank abundance distributions between eCO_2_ and aCO_2_ rings at GiFACE. AM fungal OTU richness is significantly increased in eCO_2_ rings compared with aCO_2_ rings (a; *p* = .01), whereas CO_2_ treatment has no effect on AM fungal community evenness (b & c; *p* = .95). In panels (a) and (b), filled triangles represent means, boxes show median values bound by 25–75 percentiles, and lines minima and maxima. Outliers are points >1.5 times the interquartile range away from the median

Expected differences in CO_2_ concentrations and any unexpected differences in soil physicochemical properties (pH, moisture, carbon, nitrate, ammonium), between aCO_2_ and eCO_2_ FACE rings, were checked using linear mixed‐effects models, with block as a random factor (see Tables S2–S5 for soil properties and CO_2_ fumigation data). We investigated the effects of eCO_2_ on AM fungal root colonization parameters (colonization frequency and intensity in the root system, arbuscule abundance) and community (OTU) evenness using linear mixed‐effects models. These models quantify the effects of eCO_2_ while controlling for differences between FACE ring pairs (blocks). Community evenness was quantified using Simpson's evenness index (see Morris et al., 2014). All root colonization variables and community evenness values were logit‐transformed to meet linear modelling assumptions (Warton & Hui, 2011). In all mixed‐effects models, CO_2_ treatment was included as a fixed effect, while spatial differences between blocks were accounted for by including block as a random effect. *p*‐values were calculated by comparing *t*‐statistics to a normal distribution (mean = 0 and *SD* = 1). To determine whether AM fungal OTU richness was affected by eCO_2_, we used a negative binomial generalized linear mixed‐effects model (GLMM). We controlled for differences in amplicon library sizes by including log (number of sequences) as the first (fixed) term in the model. This approach to dealing with heterogeneity in sequencing effort is advocated (Warton, Foster, De'ath, Stoklosa, & Dunstan, 2015) as it avoids many of the undesirable data properties introduced by alternatives such as rarefaction (McMurdie & Holmes, 2014). All other fixed and random effects were specified as described previously. For the purpose of visualizing OTU richness and evenness differences between treatments, we rarefied communities to the smallest library size before producing Figure 1a,b.

Differences in overall AM fungal community composition were visualized using nonmetric multidimensional scaling (NMDS) on Bray–Curtis distances. To investigate how the abundance of AM fungal OTUs changed between aCO_2_ and eCO_2_ conditions and FACE ring pairs (blocks), multivariate generalized linear models (MV‐GLMs) were used (Wang, Naumann, Wright, & Warton, 2012). A negative binomial error family was used to account for overdispersion, a common property of microbial count data. The effect of heterogeneity in sequencing depth was incorporated by including an offset term (log‐transformed number of sequences). As described above, including the interaction between block and CO_2_ allowed us to determine whether the influence of CO_2_ varied between spatial blocks. The influence of sampling date on the relationship between CO_2_ conditions and AM fungal OTU abundances was examined by repeating this analysis with the temporal samples, where we used sampling time as a factor in place of block. Sampling date was incorporated into the MV‐GLMs as a separate model covariate and as an interaction term with CO_2_ treatment. Multivariate and unadjusted univariate *p‐*values were obtained by Wald tests, both using 10,000 Monte Carlo permutations. For all MV‐GLM analyses, only OTUs that occurred in two or more samples were included, as OTUs occurring in only one sample are not likely to yield useful or statistically robust insight into the effects of eCO_2_ on AM fungal communities. For some OTUs, we observed complete separation, a problem that can cause model optimizers to crash when one or more levels of a categorical variable perfectly predict a mean abundance of 0. We identified these OTUs by their disproportionately large standard errors (e.g., >100 on the log‐link scale). As model coefficients, and by extension the univariate *p* values, are not robust when separation is observed, we remodelled these OTUs using bias‐reduction binomial GLMs (BR‐GLMs, Firth, 1993), a method that allows robust estimation of model coefficients when separation is present. As models estimate the probability of occurrence under the different CO_2_ conditions rather than actual abundance per se, we do not present output from these models in our figures.

All statistical, diversity and community analyses were conducted using the r statistical language version 3.3.1 with standard R libraries (R Core Team, 2016); the community analysis specific package “vegan” (Oksanen et al., 2018) and statistical packages “stats” (R Core Team, 2016), “mvabund” (Wang et al., 2012), “brglm” (Kosmidis, 2017) and “lme4” (Bates, Mächler, Bolker, & Walker, 2015).

## 3 RESULTS

### 3.1 GiFACE long‐term grassland experiment

The GiFACE experiment maintained significantly increased (ca. 20%) CO_2_ concentrations in eCO_2_ treatment rings, compared with aCO_2_ rings during each of the three sampling months (Table S4; May 2013, *t* = 44.26, *p* < .001; July 2013, *t* = 42.67, *p* < .001; September 2013, *t* = 84.97, *p* < .001). Importantly, soil moisture (Table S5), pH, amount of NH_4_, NO_3_, % N, % C and C/N (Table S2) did not significantly differ between eCO_2_ and aCO_2_ rings (moisture, *t* = 1.43, *p* = .15; pH, *t* = 0.00, *p* = 1; NH_4_, *t* = −.21, *p* = .83; NO_3_, *t* = −0.36, *p* = .72; % N, *t* = −1.06, *p* = .29; % C, *t* = 0.28, *p* = .78; C/N, *t* = −0.50, *p = *.62). Despite the increased concentrations of CO_2_, patterns of AM fungal root colonization did not significantly differ between eCO_2_ and aCO_2_ rings, when evaluated using the spatial samples (Table 1; *t* < 1.59, *p* > .1 in all cases).

**Table 1:** The effects of elevated CO_2_ on mycorrhizal fungal root colonization analysed with linear mixed‐effects models

Variable	Ring	Ambient	Elevated	eCO_2_ coefficient	*t*‐statistic	*p* value
% a	1	26.18 ± 14.48	20.69 ± 9.00	0.05 (0.27)	0.19	.85
	2	24.18 ± 15.09	28.16 ± 16.41			
	3	12.55 ± 14.94	15.29 ± 13.61			
% A	1	7.35 ± 5.54	5.34 ± 3.70	0.18 (0.21)	0.83	.41
	2	4.62 ± 3.65	7.87 ± 5.30			
	3	2.99 ± 4.35	3.32 ± 3.15			
% F	1	90.39 ± 8.43	89.17 ± 14.99	0.56 (0.35)	1.59	.11
	2	85.92 ± 16.21	95.42 ± 6.65			
	3	84.73 ± 9.09	87.92 ± 12.72			
% m	1	27.68 ± 5.76	27.44 ± 8.19	0.16 (0.10)	1.56	.12
	2	21.69 ± 5.89	28.83 ± 5.49			
	3	19.95 ± 7.18	22.06 ± 7.29			
% M	1	25.30 ± 6.89	25.00 ± 9.77	0.21 (0.14)	1.51	.13
	2	19.23 ± 7.64	27.65 ± 6.33			
	3	17.40 ± 7.84	19.96 ± 8.28			

Note

Estimation of mycorrhizal fungal colonization according to (Trouvelot et al., 1986) calculated for arbuscule abundance in mycorrhizal parts of root fragments (% a), arbuscule abundance in the root system (% A), frequency of mycorrhiza in the root system (% F), intensity of the mycorrhizal fungal colonization in the root fragments (% m) and intensity of the mycorrhizal fungal colonization in the root system (% M). Parameters are calculated from eight replicate samples (30 root fragments each). Mean values ± 1 *SD* of the mean are presented. For each colonization variable, ring was specified as a random intercept to account for spatial autocorrelation. A positive eCO_2_ coefficient indicates greater mycorrhizal fungal colonization under elevated CO_2_.

### 3.2 AM fungal diversity at GiFACE

We sampled a total of 55 AM fungal OTUs from eight AM fungal families across the GiFACE experiment (Figure S2). Seventy‐five samples (out of 84) successfully amplified the targeted SSU rRNA gene and, after stringent quality control (quality trimming, error correction and paired‐end alignment removed 2.53 million reads, stringent length filtering discarded a further 6.61 million reads, and 70,649 non‐AM fungal reads were removed), produced the 4,997,208 Illumina HiSeq reads (median length = 549, IQR = 1) on which our analyses are based. Visual inspection of rarefied OTU accumulation curves confirmed OTU accumulation had plateaued in all samples (HiSeq reads per sample: min = 6,454; median = 64,286; max = 245,700). The most abundant ten OTUs represented a total of 90.5% of all detected AM fungal sequences (Figure S2). Among these, OTU1 (*Rhizophagus fasciculatus*/*intraradices*/*irregularis* clade, most similar to MaarjAM VT113) and OTU4 (Glomeraceae, most similar to MaarjAM VT163) are the two most abundant OTUs in the GiFACE experiment and are also the two most abundant in both eCO_2_ and aCO_2_ rings. The rank relative abundance distributions of the ten most abundant OTUs from eCO_2_ and aCO_2_ rings were almost identical, other than for OTU305 and OTU1835 which decreased by 2 and 9 places, respectively, under eCO_2_ conditions.

### 3.3 Impacts of eCO_2_ on AM fungal communities

Based on the samples collected on 8 May (intensive spatial sampling), eCO_2_ significantly increased AM fungal OTU richness per sample over aCO_2_ (Figure 1; coefficient = 0.16, *z* = 2.57, *p* = .01), but had no significant effect on community evenness (Figure 1; coefficient = −0.003, *t* = −0.22, *p* = .95).

### 3.4 Impacts of eCO_2_ on AM fungal community composition

**Figure 2:**
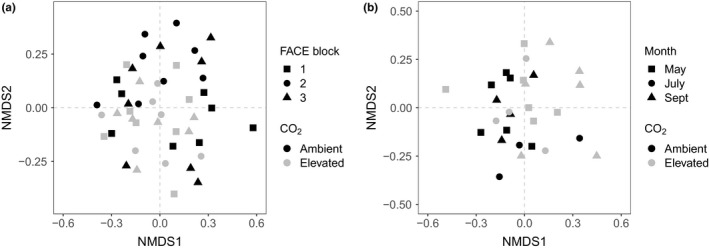
Nonmetric multidimensional scaling (NMDS) plots for (a) spatial and (b) temporal samples from GiFACE. Black and grey symbols represent samples from aCO_2_ and eCO_2_ treatments, respectively. Square, circle and triangle symbols either represent samples from FACE ring blocks 1, 2 and 3, respectively (a), or samples collected in May, July and September 2013, respectively (b). Samples that appear closer together represent AM fungal communities that are compositionally more similar than those further apart

Compositional changes in AM fungal communities were visualized using nonmetric multidimensional scaling plots (NMDS) based on Bray–Curtis distance (Figure 2a; stress = 0.23). There was no clear separation between aCO_2_ and eCO_2_ AM fungal communities in the spatial sampling, while in our temporal sampling, AM fungal communities from aCO_2_ and eCO_2_ show minor differences in composition.

**Figure 3:**
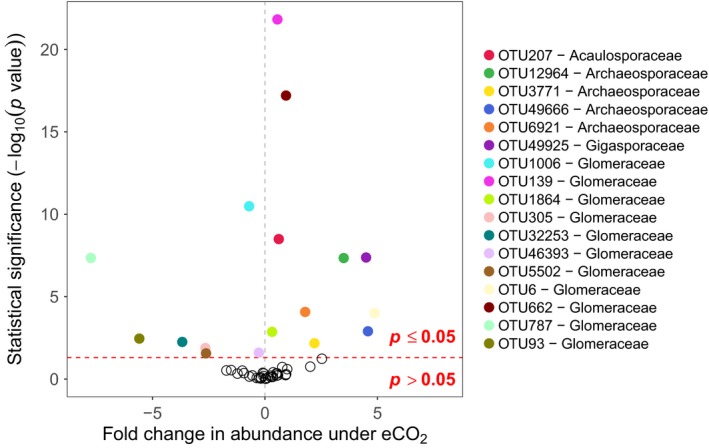
Volcano plot of MV‐GLM modelled shifts in OTU abundances under eCO_2_ conditions, compared to aCO_2_ conditions. Coloured points above the horizontal dotted line represent OTUs that showed statistically significant shifts in abundance (*p* < .05), whereas hollow points below the horizontal dotted line represent OTUs that did not show statistically significant shifts (*p* > .05). OTUs further to the left were less abundant under eCO_2_ conditions, while OTUs to the right were more abundant under eCO_2_ conditions

A more detailed examination of AM fungal community composition by modelling OTU abundances using multivariate models (MV‐GLMs or BR‐GLMs) of spatial sample data revealed that eCO_2_ treatment (*z*_1, 44_ = 11.50, *p* < .001), FACE block (*z*_2, 43_ = 18.91, *p* < .001) and their interaction (*z*_2, 43_ = 13.63, *p* < .001) significantly affected OTU abundances, after controlling for unequal sequencing depths. Univariate tests showed that 17 OTUs were significantly affected by eCO_2_ regardless of FACE ring (Figure 3; Table S6; *z*_1, 44_ > 0.0003, *p* < .041 in all cases), and 22 OTUs were significantly affected by the interaction of eCO_2_ and FACE ring together (Figure 3; Table S6; *z*_2, 43_ > 2.89, *p* < .049 in all cases). Of the OTUs affected by CO_2_ treatment, seven were significantly less abundant under eCO_2_ conditions than aCO_2_ conditions (Figure 3; OTUs 93, 305, 787, 1,006, 5,502, 32,253, and 46,393; *z*_1, 44_ > 0.0003, *p* < .027 in all cases), whereas 10 OTUs were significantly more abundant under eCO_2_ than aCO_2_ conditions (Figure 3; OTUs 6, 139, 207, 662, 1,864, 3,771, 6,921, 12,964, 49,666 and 49,925; *z*_1, 44_ > 0.035, *p* < .041 in all cases). For the OTUs that showed a mean abundance of 0 under certain treatments, BR‐GLMs showed that: OTU 49,666 had an overall positive response to elevated CO_2_ (coef = 4.58, *z*_1, 44_ = 3.23, *p* < .01), therefore being more likely to occur in elevated CO_2_ conditions. In contrast, OTU 787 was significantly less likely to occur in eCO_2_ conditions (coef = −7.74, *z*_1, 44_, *p* < .001). OTUs 277 and 43,243 showed significant interactions between block and CO_2_ conditions, indicating that CO_2_ conditions only influenced their probability of occurrence in specific blocks (|*z*_2, 43_| > 2.47, *p* < .05 in both cases).

**Figure 4:**
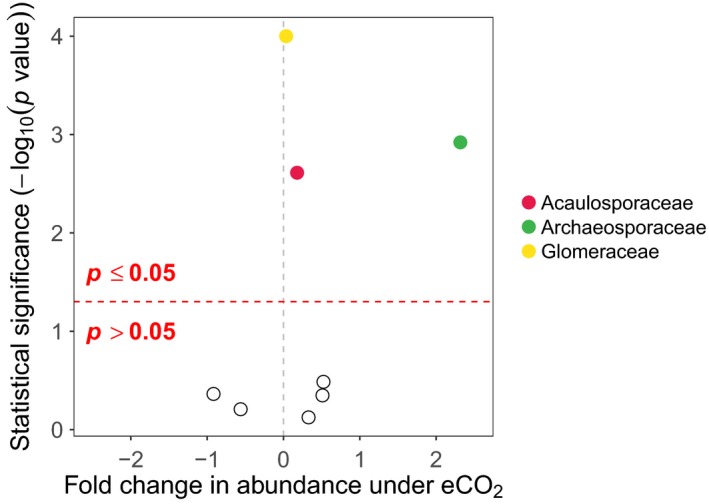
Volcano plot of MV‐GLM modelled shifts in AM fungal family abundances under eCO_2_ conditions, compared to aCO_2_ conditions. Coloured points above the horizontal dotted line represent families that showed statistically significant shifts in abundance (*p* < .05), whereas hollow points below the horizontal dotted line represent families that did not show statistically significant shifts (*p* > .05). Families further to the left were less abundant under eCO_2_ conditions, while families to the right were more abundant under eCO_2_ conditions

Univariate tests showed that two families (Archaeosporaceae and Glomeraceae) were significantly affected by eCO_2_ (Figure 4; Table S7) and were more abundant under eCO_2_ than aCO_2_ conditions (*z*_1, 44_ > 3.25, *p* < .0008 in both cases). The same families also had significantly different abundances across FACE rings, but only the Glomeraceae were significantly affected by the interaction between FACE rings and eCO_2_ (*z*_2, 43_ > 13.27, *p* < .0001). Acaulosporaceae and Ambisporaceae both showed evidence of separation issues for several covariates (*SE* > 300 for some factor levels). Modelling these families with BR‐GLMs showed that Ambisporaceae were not significantly more likely to occur in any of the treatments or blocks (|*z|* < 1.09, *p* > .28 for all terms), whereas Acaulosporaceae were significantly more likely to occur under eCO_2_ conditions (coef = 0.18, *z*_1, 44_ = 3.03, *p* < .01), but also showed significant spatial variation between blocks (|*z*|_2, 43_ = 4.74, *p* < .001 for all block terms).

### 3.5 Temporal changes in AM fungal communities

**Figure 5:**
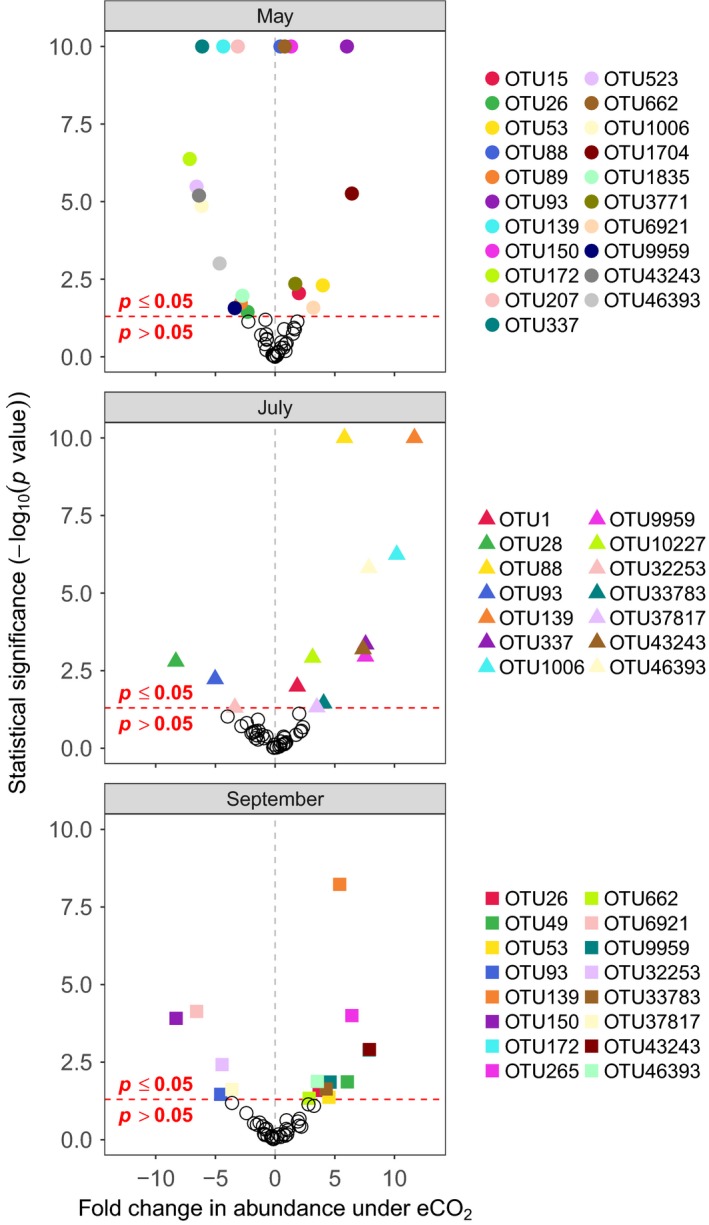
Volcano plot of MV‐GLM modelled shifts in OTU abundances under eCO_2_ conditions and between sampling months. Coloured points above the horizontal dotted line represent OTUs that showed statistically significant shifts in abundance (*p* < .05), whereas hollow points below the horizontal dotted line represent OTUs that did not show statistically significant shifts (*p* > .05). OTUs further to the left were less abundant under eCO_2_ conditions, while OTUs to the right were more abundant under eCO_2_ conditions. For OTUs where *p* < 10^–10^, we rounded *p* to 10^–10^ for ease of visualization

Arbuscular mycorrhizal fungal OTU richness did not change significantly across sampled months or between aCO_2_ and eCO_2_ treatments over the course of the temporal data set (GLMM; coefficient = 0.02, *z* = 0.02, *p* = .89; Table S8). Nor was there any significant interaction between sampling date (i.e., month) and CO_2_ treatment (*z *> −0.31, *p* > .76 for all interaction terms). Compositional changes in AM fungal communities, visualized using nonmetric multidimensional scaling plots (Figure 2b), showed a minor separation between AM fungal communities from aCO_2_ and eCO_2_ through time (Figure 2b, stress = 0.20). Analysis of the temporal data set using MV‐GLMs revealed that sampling time (*z*_2, 26_ = 14.44, *p* < .001), FACE ring (*z*_2, 24_ = 22.85, *p* < .001) and CO_2_ treatment (*z*_1, 23_ = 12.45, *p < *.001) all affected OTU abundances alone, as well as the interaction between sampling time and CO_2_ treatment (*z*_2, 21_ = 12.35, *p < *.001). Twenty‐seven OTUs were found to significantly differ in abundance between sampling time (Figure 5 and Table S9; *z*_2, 27_ > 0.003, *p* < .049 in all cases), 34 OTUs differed between FACE blocks (Figure 5 and Table S9; *z*_2, 27_ > 0.06, *p* < .043 in all cases) and 21 OTUs differed between CO_2_ treatment (Figure 5 and Table S9; *z*_1, 27_ > 0.0003, *p* < .039 in all cases). Additionally, 22 OTUs had a significant interaction term between sampling time and CO_2_ treatment indicating that the effect of eCO_2_ on AM fungal populations may vary throughout the period of the year we sampled (Figure 5 and Table S9; *z*_2, 27_ > 0.004, *p* < .05 in all cases).

**Figure 6:**
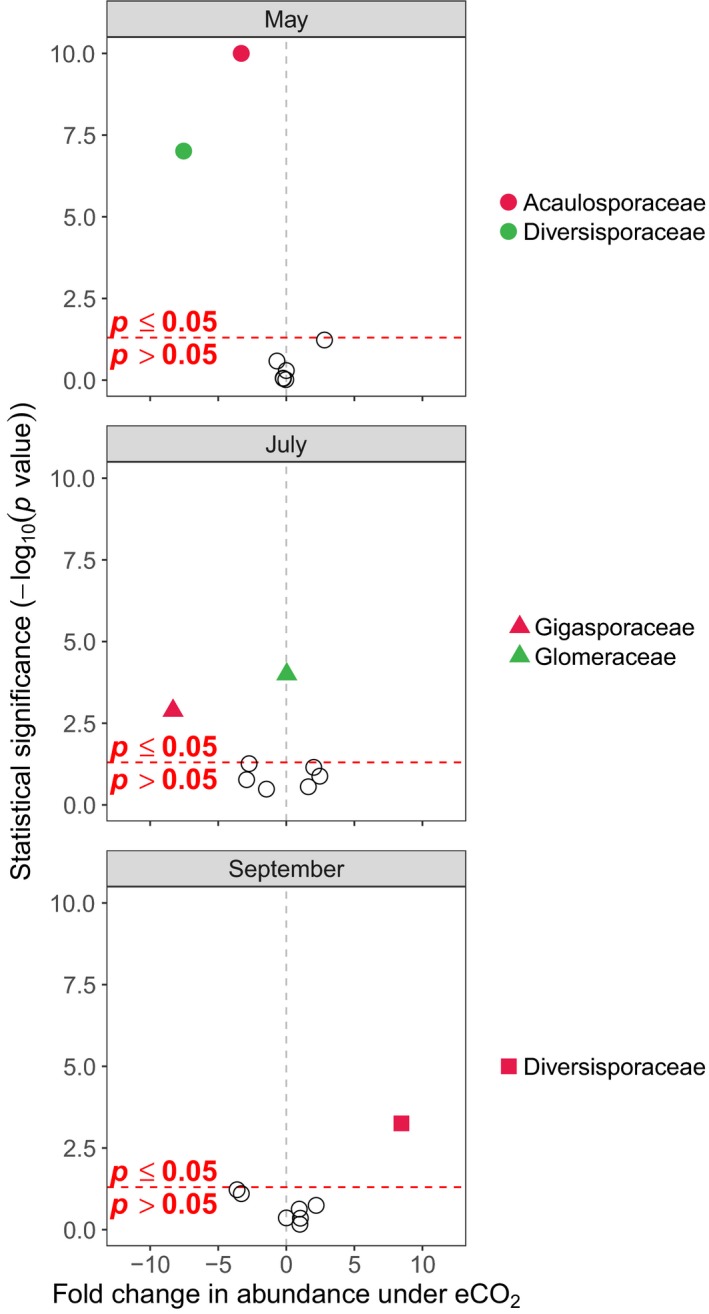
Volcano plot of MV‐GLM modelled shifts in AM fungal family abundances under eCO_2_ conditions and between sampling months. Coloured points above the horizontal dotted line represent families that showed statistically significant shifts in abundance (*p* < .05), whereas hollow points below the horizontal dotted line represent families that did not show statistically significant shifts (*p* > .05). Families further to the left were less abundant under eCO_2_ conditions, while families to the right were more abundant under eCO_2_ conditions. For families where *p* < 10^–10^, we rounded *p* to 10^–10^ for ease of visualization

All of the AM fungal families significantly differed in abundance or occupancy across sampling times (Figure 6 and Table S10; *z*_2, 26_ > 0.043, *p* < .01 in all cases), except the Claroideoglomeraceae (*z*_2, 26_ < 0.38, *p* > .71 for all sampling months), Gigasporaceae (*z*_2, 26_ < 0.54, *p* > .06 for all sampling months) and the Paraglomeraceae (*z*_2, 26_ < 0.55, *p* > .30). In addition, the occupancy of two AM fungal families was significantly lower in eCO_2_ rings compared with aCO_2_ rings (BR‐GLM; Acaulosporaceae, *z*_1, 27_ = −30.04, *p* < .001; Diversisporaceae, *z*_1, 27_ = −5.33, *p* < .001), across sampling months. Three AM fungal families (Diversisporaceae, Gigasporaceae and Glomeraceae) also had significant interaction terms (*z*_2, 21_ > 0.03, *p* < .004 for all cases), indicating that the effect of eCO_2_ on some AM fungal families may vary temporally.

## 4 DISCUSSION

This study reveals the ecological response of AM fungal communities from an old‐grown semi‐natural temperate grassland to long‐term (>15 years) eCO_2_ (ca. +20% of ambient) conditions that reflect future climate change scenarios. While root colonization by AM fungi did not change in response to eCO_2_ (Table 1), the AM fungal community did significantly increase in taxon (OTU) richness, but with no change in community evenness, under eCO_2_ treatments (Figure 1). In addition, the composition of AM fungal communities also significantly responded to eCO_2_ treatments. However, this was driven by subtle responses of specific AM fungal taxa (OTUs), with these populations both increasing and decreasing in abundance in response to eCO_2_ (Figures 3 and 5), rather than a complete turnover or resorting of all AM fungal taxa (Figure 2). Furthermore, these population‐level responses varied through time with population dynamics significantly interacting with eCO_2_ responses. These responses were observed both at the species (OTU) level and at the family level, with the highly dominant Glomeraceae, significantly increasing in abundance under eCO_2_ (Figure 4), which may be reflective of the ecologically relevant functional traits (e.g., mycelium growth, phosphate and carbon metabolism, and spore formation) that are conserved at higher taxonomic levels within the AM fungi (Cotton et al., 2015; Powell et al., 2009).

### 4.1 Root colonization with AM fungi

In contrast to our expectations that AM fungal root colonization would increase under eCO_2_, there was no significant increase observed in the mixed roots sampled from eCO_2_ treatments compared with aCO_2_ treatments. Plant roots in natural grasslands are usually highly colonized with AM fungi (Smith & Read, 2008), and the frequency of mycorrhization within roots at GiFACE was close to saturation regardless of CO_2_ treatment (Table 1). Thus, further increases under eCO_2_ conditions could be limited. While previous research has shown increased mycorrhizal fungal colonization under eCO_2_ (see Alberton et al., 2005 for meta‐analyses; Treseder, 2004), the experimental designs behind these results have varied extensively (e.g., with different: fumigation approaches, CO_2_ concentrations, plant species, duration and fertilizer treatments) and very few studies have assessed root colonization from FACE experiments (but see Gamper et al., 2004; Garcia, Ovasapyan, Greas, & Treseder, 2008; Runion et al., 1994; Staddon, Gregersen, & Jakobsen, 2004). Typically, FACE studies recorded far smaller responses of AM fungal root colonization to eCO_2_ than all other experimental setups (e.g., pots and fumigation chambers; for review, see Alberton et al., 2005; Treseder, 2004), suggesting results may be dependent upon the environmental context, and/or temporal and spatial scale of the study. Pot‐based eCO_2_ fumigation experiments are limited by cultivable (usually fast‐growing) AM fungal taxa. They are typically of short duration and mycorrhizal networks develop during the experiment, with assessments of root colonization reflecting increased colonization speed under eCO_2_ (Gamper et al., 2004; Treseder, 2004). Within FACE experiments, root colonization by AM fungi tends to be evaluated after the experiment is well established, reflecting end‐point responses to eCO_2_ conditions (Gamper et al., 2004). Thus, the long‐term duration of GiFACE coupled with the natural diversity of grassland plant and AM fungal taxa has allowed for similar levels of root colonization to exist regardless of CO_2_ treatment.

### 4.2 AM fungal diversity in GiFACE

We recorded a total of 55 AM fungal taxa (OTUs) from eight AM fungal families. This estimate of AM fungal OTU richness is as expected from grassland systems, given both the number and nature (i.e., mixed roots) of samples examined, and the NGS methods used (Dumbrell et al., 2011; Hiiesalu et al., 2014; Moora et al., 2014). Average AM fungal richness was higher under eCO_2_ compared with aCO_2_ treatments (though both treatments had equivalent total taxon pools of 51 OTUs). Previously observed increases in total fungal diversity under eCO_2_ are determined by the length of the experiment (Veresoglou et al., 2016). Longer‐term studies allow greater recruitment from the meta‐community of taxa pre‐adapted to these new environmental conditions and if sufficiently long term, the evolution of new species (Johnson et al., 2013; Veresoglou et al., 2016), both of which increase local taxa richness. At the GiFACE, the expansive semi‐natural grassland surrounding eCO_2_ FACE rings and the long‐term experimental duration certainly provide ample opportunity for recruitment of pre‐adaptive AM fungal taxa from the meta‐community. However, as few OTUs were unique to eCO_2_ treatments, it may be that greater resource (rhizodeposited photosynthates; Cheng et al., 2012; Drigo et al., 2010, 2013) and/or habitat (increased root biomass; Canadell, Pitelka, & Ingram, 1996; Carrillo et al., 2014; Fitter et al., 1997) availability is simply supporting a greater number of AM fungal OTUs than in aCO_2_ treatments.

The indirect influence of eCO_2_ on AM fungi via increases in root biomass (Canadell et al., 1996; Carrillo et al., 2014; Fitter et al., 1997) and/or root turnover (Allard et al., 2005; Canadell et al., 1996; Fitter et al., 1997; Nie, Lu, Bell, Raut, & Pendall, 2013) are potentially major factors explaining our results. If increases in rhizodeposited photosynthates in eCO_2_ treatments were driving changes in AM fungal communities, this change in resource limitation between treatments would likely reduce the evenness of AM fungal communities, as initially hypothesized. However, there was little evidence of this, and AM fungal communities from both aCO_2_ and eCO_2_ treatments displayed typical patterns of dominance (ca. >40% sequences belong to the dominant OTU) found consistently across AM studies (Dumbrell, Nelson, Helgason, Dytham, & Fitter, 2010b; Lekberg et al., 2012; Verbruggen, van der Heijden, Weedon, Kowalchuk, & Rö‐Ling, 2012). As increases in root biomass under eCO_2_ are driven by increases in root turnover and the production of fine roots (Allard et al., 2005; Canadell et al., 1996; Fitter et al., 1997; Nie et al., 2013), which are preferentially colonized by AM fungi over older roots (Smith & Read, 2008), this offers a parsimonious explanation for observed results. Moreover, data from GiFACE demonstrate increases in fine root production and root turnover in eCO_2_ treatments (Lenhart, 2008).

### 4.3 AM fungal community composition

The composition of AM fungal communities significantly changes in response to eCO_2_ at GiFACE. However, this reflects subtle changes in the relative abundances of specific AM fungal populations and not the presence of a novel eCO_2_ community that is entirely compositionally distinct from communities recorded in aCO_2_ conditions. These findings support our initial hypothesis and result from earlier work on agricultural systems (Cotton et al., 2015) and pot‐based experiments (Klironomos et al., 2005; Klironomos, Ursic, Rillig, & Allen, 1998). However, whether these population‐level responses reflect predicted shifts towards faster growing r‐strategists (Glomeraceae) at the expense of slower growing K‐strategists (Gigasporaceae) is less clear.

At the family level, the Glomeraceae did significantly increase in eCO_2_ treatments compared with aCO_2_ treatments, while the Gigasporaceae did not change significantly. Cotton et al. (2015) reported similar results from an agricultural FACE experiment. This highlighted that the easily cultured Glomeraceae taxa that are most likely r‐strategists could grow and provide phosphorus to host plants quickly (Boddington & Dodd, 1999; Sýkorová, Ineichen, Wiemken, & Redecker, 2007) with a high reciprocal capability of using recently fixed carbon (Drigo et al., 2010), whereas slower growing K‐strategists like taxa of Gigasporaceae would be outcompeted (Cotton et al., 2015). However, at the species level, Glomeraceae OTUs increased, decreased and did not change in abundance in response to eCO_2_, suggesting this mechanism is not universally applicable across AM fungal taxa within a family. For example, the commonest OTU (OTU1) at GiFACE was from the Glomeraceae and did not respond differently to eCO_2_ and aCO_2_ treatments. This taxon was putatively identified as *Rhizophagus fasciculatus*/*intraradices/irregularis* cluster (VT113; following Öpik et al., 2010; Öpik et al., 2013), which is a widely distributed AM fungal taxon, found throughout environmental studies and as cultures (Öpik et al., 2010; Savary et al., 2018). OTU1's (VT113) lack of response to eCO_2_ treatment was surprising as its sister taxon (VT114) is considered to be particularly effective at quickly obtaining carbon from host plants (Vandenkoornhuyse et al., 2007) and subsequently tends to increase in abundance following eCO_2_ exposure (Cotton et al., 2015). Very little is known about the mechanisms and regulation of resource exchange between plants and AM fungi (Cotton et al., 2015) and while the principle of “optimal allocation” (Johnson et al., 2013) has been suggested as an explanation behind increased abundance of r‐strategists in the eCO_2_ world, not enough information is available to separate all AM fungal taxa into r and K groupings. Indeed, similar patterns were observed across both AM fungal families (Archaeosporaceae and Glomeraceae) that increased in abundance under eCO_2_ conditions. Across these families, approximately half the OTUs decreased and the rest increased in abundance in response to eCO_2_ (Figure 3, Table S6). Generally, those OTUs increasing in abundance were present but exceptionally rare within aCO_2_ treatments, suggesting that taxa which normally exist at the edge of their realized niche may benefit from an eCO_2_ fertilization effect.

### 4.4 Temporal changes in AM fungal communities

Across temporal samples collected in May, July and September, the composition of AM fungal communities significantly changed in response to eCO_2_, and again this reflected changes in the abundances of a few populations rather than a larger resorting of the AM fungal community. There was a strong interaction between CO_2_ treatments and sampling month, and the populations that changed in density in response to eCO_2_ were not consistent through time. Notably, ca. 50% of all OTUs that responded to eCO_2_ did not do so in all sampled months. Moreover, only eight OTUs, which showed a significant response to eCO_2_ from the main spatially extensive samples, still showed a response when considered temporally, and in some cases (e.g., OTU 93), the overall direction of their response (i.e., increased or decreased abundance) had reversed.

In temperate European grasslands, during the main plant growth period (April to October), AM fungal communities vary little in composition (Davison et al., 2012; Dumbrell et al., 2011; Santos‐González, Finlay, & Tehler, 2007; Varela‐Cervero, López‐García, Barea, & Azcón‐Aguilar, 2016). Similar results were observed at GiFACE, with only subtle changes in overall AM fungal community composition during this period. However, some AM fungal populations did show significant changes in abundance between samples regardless of CO_2_ treatment, and a number of these had their temporal dynamics altered by eCO_2_ treatments. Abiotic soil factors are consistent across GiFACE treatments (Table S2), as is temporal variability in those linked to climate such as soil moisture (Table S2) and temperature. Thus, changes in temporal dynamics of AM fungal populations between aCO_2_ and eCO_2_ treatments are most likely caused by changes in biotic variables that are known to be influenced by eCO_2_. As root biomass and associated increases in root turnover (births and deaths) accumulate earlier in the growing season under eCO_2_ (Fitter et al., 1997), temporal changes in the abundance of AM fungal populations (i.e., population phenology) may simply be happening earlier. These temporal changes would also be evident in aCO_2_ treatments, but lagging behind those observed under eCO_2_. While formally testing this would require far greater temporal sampling than presented in this work, our preliminary observations suggest this is not the case as population dynamics from aCO_2_ treatments are largely unpredictable from eCO_2_ data and vice versa. Alternatively, the continued temporal turnover of roots and increased accumulation of root biomass under eCO_2_ conditions (Allard et al., 2005; Canadell et al., 1996; Nie et al., 2013) is simply providing a dynamic source of novel environments for the AM fungi. This would allow rarer and potentially weaker competitors a chance to establish in roots in the absence of other AM fungi. If this were the case, the within‐plant‐growth‐period dynamics of many AM fungal populations would exhibit largely stochastic fluctuations in population abundances, and not a clear unimodal relationship between sampling date and population abundance. While there is evidence for this within the GiFACE data, finer scale temporal sampling is required to demonstrate this conclusively.

## 5 CONCLUSION

This work demonstrates the importance of long‐term experiments in understanding the impact of global change on terrestrial ecosystems (Maček, Vodnik, Pfanz, Low‐Décarie, & Dumbrell, 2016) and the need to account for both aboveground and belowground components of biodiversity if we are to fully understand how these ecosystems will behave in the future. The study provides a comprehensive assessment of how AM fungi from semi‐natural grasslands respond to eCO_2_ and uses the most recent molecular tools to probe the longest running temperate grassland FACE experiment globally. Results highlight the susceptibility of these functionally important soil microbes to global change, with responses evident across both population and community levels. Importantly, we demonstrate that the dynamics of AM fungal populations with the main plant growth period change in response to eCO_2_. This raises critical questions and highlights the need for far greater research into temporal response of AM fungi to environmental change; the functional differentiation observed across AM fungal taxa means any changes in their temporal dynamics has the potential to resonate throughout associated plant communities, changing aboveground competition dynamics and thus future ecosystem productivity in currently unpredictable ways.

## AUTHOR CONTRIBUTION

The authors declare that there are no conflicts of interest. I.M., A.J.D., D.V. and C.M. designed the research; I.M., N.Š., D.V., G.M. and C.M. performed the research (field and laboratory work); A.J.D., N.Š. and D.R.C. analysed the data; and I.M., A.J.D., N.Š. and D.R.C. wrote the manuscript.

## DATA AVAILABILITY

Raw sequence data have been submitted to the European Nucleotide Archive under the Accession no. PRJEB19402. Other data necessary to reproduce our analyses are available in Supporting Information Tables[Link], [Link], [Link], [Link], [Link].
