# Supplementary material for: Impacts of long‐term elevated atmospheric CO2 concentrations on communities of arbuscular mycorrhizal fungi
Source: Mol Ecol. 2019 Jul 17;28(14):3445–58. doi: 10.1111/mec.15160 (PMC6851679; doi:10.1111/mec.15160)
Supplement: Supplementary file 1 [file MEC-28-3445-s001.pdf]

Impacts of long-term elevated atmospheric CO_2 concentrations on communities of arbuscular mycorrhizal fungi

Irena Maček [1, 2, *], Dave R. Clark [3], Nataša Šibanc [1, 2, 4], Gerald Moser [5], Dominik Vodnik [1], Christoph Müller [5, 6] and Alex J. Dumbrell [3, *]

[1] *Biotechnical Faculty, University of Ljubljana, Jamnikarjeva 101, SI-1000 Ljubljana, Slovenia*
[2] *Faculty of Mathematics, Natural Sciences and Information Technologies (FAMNIT), University of Primorska, Glagoljaška 8, SI-6000 Koper, Slovenia*
[3] *School of Biological Sciences, University of Essex, Wivenhoe Park, Colchester. CO4 3SQ. United Kingdom*
[4] *Slovenian Forestry Institute, Večna pot 2, SI-1000 Ljubljana, Slovenia*
[5] *Department of Plant Ecology, Justus-Liebig University Giessen, Heinrich-Buff-Ring 26, 35392 Giessen, Germany*
[6] *School of Biology and Environmental Science and Earth Institute, University College Dublin, Belfield, Dublin 4, Ireland*

*Correspondence:
Irena Maček, tel. +386 (0)1320 3244, fax: +386 (0)1423 1088, e-mail: irena.macek@bf.uni-lj.si
Alex J. Dumbrell, tel. +44 (0)1206 87 2539, fax: +44 (0)1206 87 2592, e-mail: adumbrell@essex.ac.uk

Supporting Information

Figure S1. GiFACE experiment spatial map. Ambient CO_2 rings (A1, A2, A3), elevated CO_2 rings (E1, E2, E3).

Figure S2. Neighbor-Joining phylogeny of AM fungal OTUs recorded at GiFACE. The phylogeny includes the sequence of closest virtual taxa (VT, MaarjAM database; Öpik et al., 2010). Bootstrap values (10,000 replicates) are shown above the branches and before the node to which they correspond. The tree is rooted with *Corallochytrium limacisporum* as a general outgroup to all fungi and *Geosiphon pyriformis* as a specific outgroup to the AM fungi. Clades indicated with numbers correspond to clusters of AM fungal morphospecies: (1) *Scutellospora aurigloba*, *S. calospora*; (2) *Funneliformis africanum*, *F. constrictus*; (3) *Funneliformis caledonius*, *F. fragilistratus*, *F. geosporus*, *F. verruculosus*, *Rhizophagus clarus*; (4) *Glomus hoi*, *G. macrocarpum*; (5) *Rhizophagus intraradices*, *R. irregularis*, *R. vesiculiferus*; (6) *Rhizophagus fasciculatus*, *R. intraradices*. Taxonomic classification is following Öpik et al. (2013), nomenclature according to Schußler & Walker (2010). Higher level taxonomic classifications are shown vertically.

Table S1. List of plant species within GiFACE rings (May 2013).

Table S2. Mean pH values, NH_4, NO_3, %N, %C and C/N from each ring plus or minus one standard deviation of the mean.

Table S3. Fumigation period in CO_2 enriched rings.

Table S4. Day time CO_2 fumigation. Mean ± standard deviations of three months for each ring. Duration times for CO_2 fumigation are in Table S3. E – elevated, A – ambient.

Table S5. Soil moisture (%) of week prior to sampling. Values are mean ± standard deviations of 7 days and 4 replicates per ring. E – elevated, A – ambient.

Table S6 (uploaded as a separate file). Results of Wald tests on multivariate models relating OTU abundances to CO_2 treatment. CO_2 coefficients are relative to the ambient treatment, meaning that a positive coefficient indicates increased abundance under elevated CO_2 and a negative coefficient, a decreased abundance under elevated CO_2.

Table S7 (uploaded as a separate file). Coefficients from multivariate GLMs and *P*-values from Wald tests relating AM fungal family abundances to CO_2 treatment. Models treated ambient samples as the reference level, meaning a positive coefficient for elevated CO_2 would indicate increased abundance relative to ambient samples.

Table S8. Negative binomial generalised linear mixed model, with sampling time*CO_2 interaction term, and block specific random intercept. May and ambient samples used as reference levels.

Table S9 (uploaded as a separate file). Coefficients from multivariate GLM models and *P*-values calculated by Wald tests on AM fungal OTU abundances in relation to CO_2 treatment and sampling date. CO_2 coefficients are relative to ambient and May samples, meaning a positive coefficient indicates an increase in mean abundance in elevated CO_2 conditions or in other months.

Table S10 (uploaded as a separate file). Coefficients from multivariate GLMs and *P*-values from Wald tests relating AM fungal family abundances to CO_2 treatment and sampling date. As before, May and ambient samples served as reference levels in the models. Therefore, a positive coefficient for September or elevated CO_2 would indicate increased abundance relative to May or ambient samples, respectively.

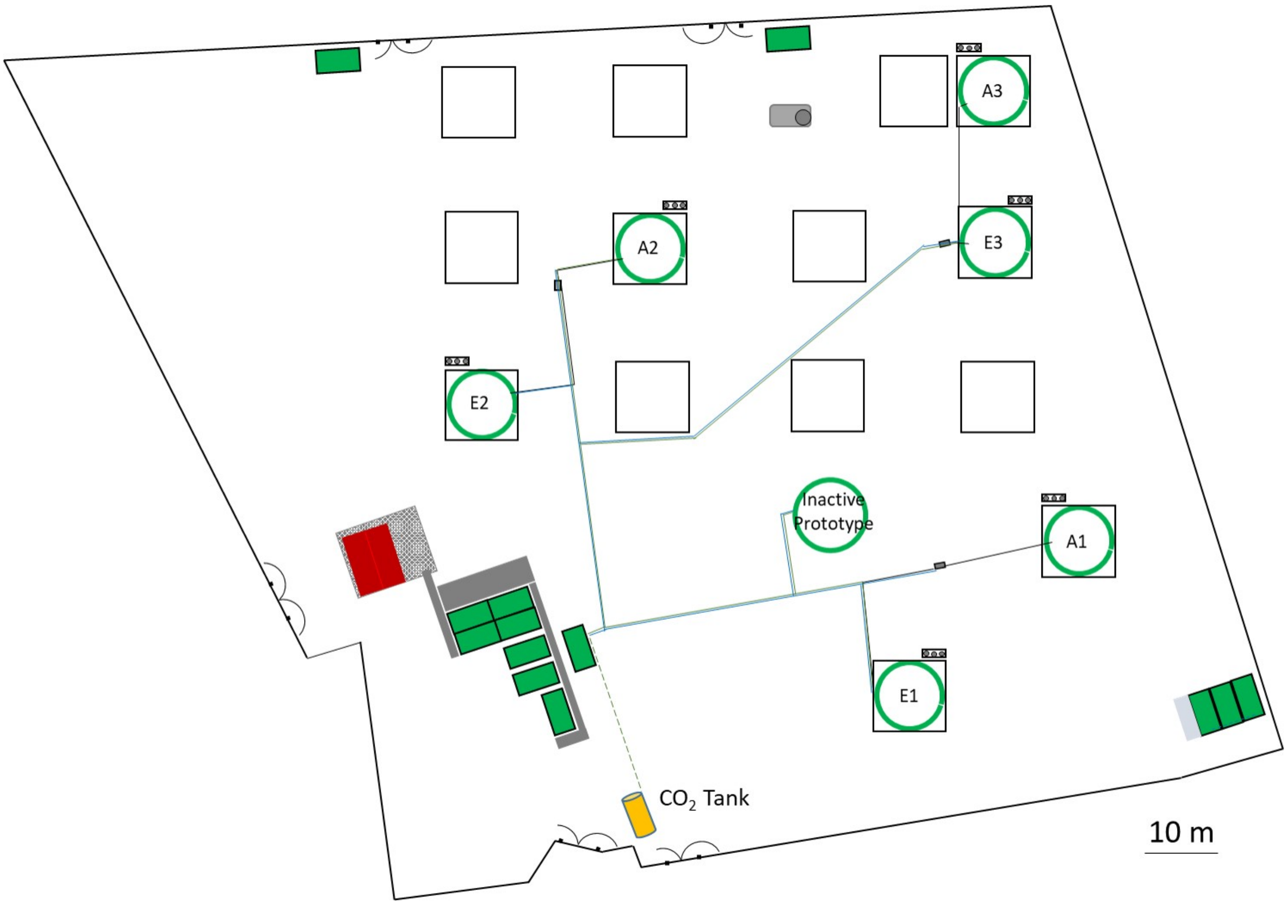

Figure S1. GiFACE experiment spatial map. Ambient CO_2 rings (A1, A2, A3), elevated CO_2 rings (E1, E2, E3).

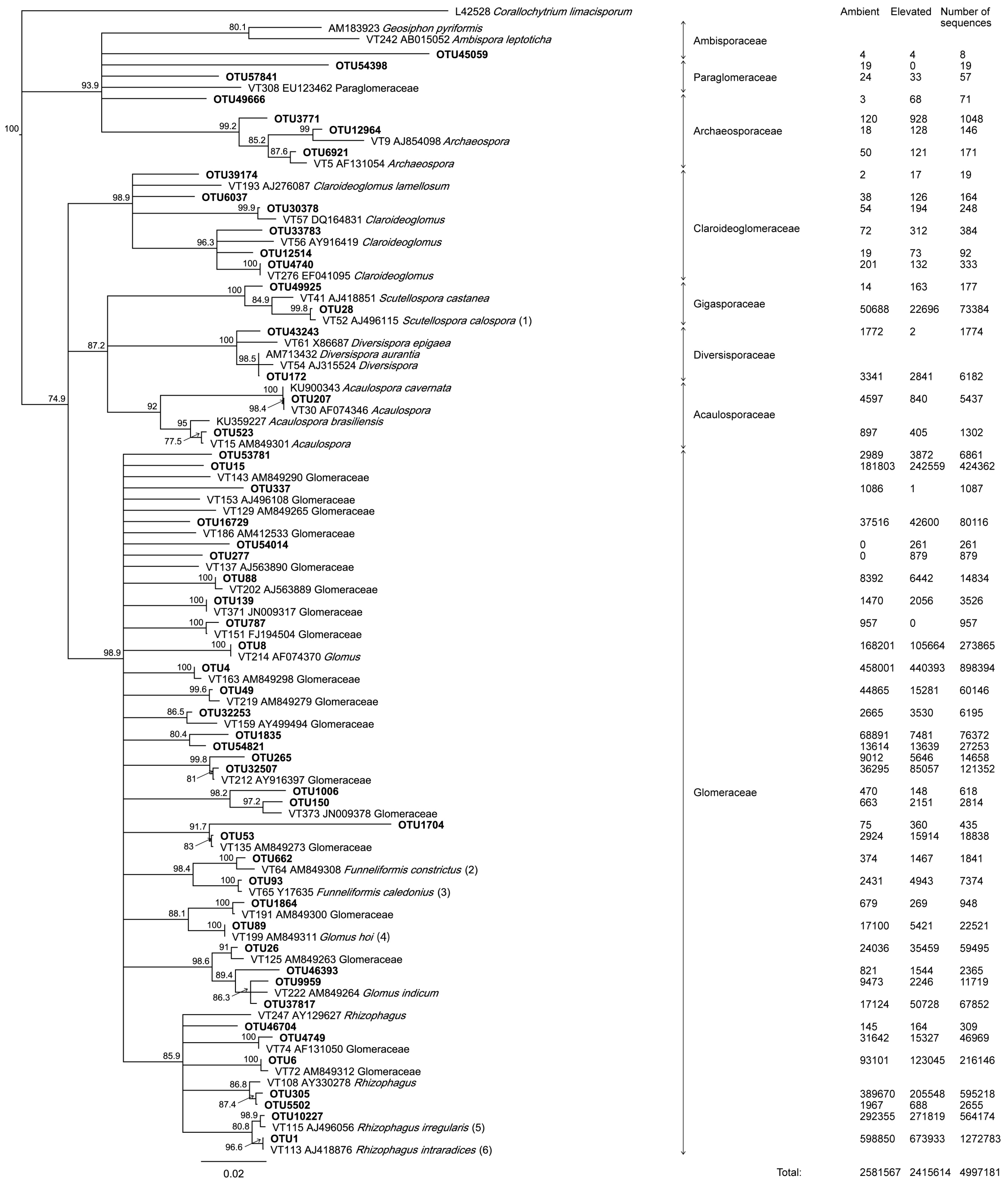

Figure S2. Neighbor-Joining phylogeny of AM fungal OTUs recorded at GiFACE. The phylogeny includes the sequence of closest virtual taxa (VT, MaarjAM database; Öpik et al., 2010). Bootstrap values (10,000 replicates) are shown above the branches and before the node to which they correspond. The tree is rooted with *Corallochytrium limacisporum* as a general outgroup to all fungi and *Geosiphon pyriformis* as a specific outgroup to the AM fungi. Clades indicated with numbers correspond to clusters of AM fungal morphospecies: (1) *Scutellospora auriglobа*, *S. calospora*; (2) *Funneliformis africanum*, *F. constrictus*; (3) *Funneliformis caledonius*, *F. fragilistratus*, *F. geosporus*, *F. verruculosus*, *Rhizophagus clarus*; (4) *Glomus hoi*, *G. macrocarpum*; (5) *Rhizophagus intraradices*, *R. irregularis*, *R. vesiculiferus*; (6) *Rhizophagus fasciculatus*, *R. intraradices*. Taxonomic classification is following Öpik et al. (2013), nomenclature according to Schußler & Walker (2010). Higher level taxonomic classifications are shown vertically.

Table S1. List of plant species within GiFACE rings (May 2013).

	Species coverage in GiFACE rings (%)					
Plant species	A1	A2	A3	E1	E2	E3
Geranium pratense	20	29	15	20	10	20
Arrhenatherum elatius	15	10	3	15	38	15
Galium mollugo	10	10	15	20	15	10
Trisetum flavescens	10	10	10	3	10	10
Poa pratensis	1	6	2	1		6
Holcus lanatus	3	3	1	6	1	3
Brachythecium rutabulum	1	3	3	3	3	3
Anthoxanthum odoratum	2	1	1	3	1	1
Lathyrus pratensis	1	1	1	3	1	2
Cerastium holosteoides	1	1	3	1	1	1
Saxifraga granulata	1	1	1	2	1	1
Sanguisorba officinalis	1	1	2	1	<1	3
Dactylis glomerata	1	1	1	1	1	1
Festuca rubra agg.	1	1	1	1	1	1
Rumex acetosa	1	1	1	1	1	1
Plantago lanceolata	1	1	1	1	1	1
Poa trivialis	<1	1	1	1	1	1
Glechoma hederacea	<1	1	1	1	1	1
Ranunculus acris		1	1		1	<1
Taraxacum officinale agg.		1	1			1
Erophila verna	1	1			<1	
Bellis perennis			1		<1	<1
Festuca pratensis		<1		1		
Bromus racemosus			1			
Medicago lupulina	1					
Avenochloa pubescens	<1		<1			
Filipendula ulmaria		<1	<1			
Cirsium oleraceum			<1			
Bromus hordeaceus			<1			
Luzula campestris				<1		
Lotus corniculatus				<1		
Trifolium pratense		<1			<1	
Alopecurus pratensis		<1				

Table S2. Mean pH values*, NH_4, NO_3, %N, %C and C/N from each ring plus or minus one standard deviation of the mean.

	ambient			elevated		
	A1	A2	A3	E1	E2	E3
pH (0.1 M KCl)	5.70 ± 0.25	6.08 ± 0.1	6.03 ± 0.17	5.73 ± 0.17	6.08 ± 0.1	6.00 ± 0.08
NH_4	2.74 ± 2.21	2.24 ± 1.89	4.63± 5.22	3.35 ± 2.85	2.76 ± 2.30	3.11 ± 2.99
NO_3	2.67 ± 3.69	3.95 ± 6.43	5.54 ± 6.87	3.50 ± 5.09	4.26 ± 6.20	3.25 ± 4.56
%N	0.37 ± 0.07	0.50 ± 0.10	0.57 ± 0.07	0.41 ± 0.09	0.46 ± 0.12	0.51 ± 0.09
%C	5.03 ± 4.24	5.56 ± 2.26	5.89 ± 0.99	4.69 ± 1.40	8.36 ± 12.93	5.40 ± 0.86
C/N	14.55 ± 15.66	11.00 ± 2.81	10.38 ± 1.62	11.29 ± 1.27	34.08 ± 8.90	10.75 ± 1.15

*Data from Jäger et al. (2003).

Table S3. Fumigation period in CO_2 enriched rings.

Month	from	until	Fumigation period
	(Time in MEZ)		(in hours)
January	11:01	14:00	3
February	10:01	15:00	5
March	9:01	16:00	7
April	8:01	17:00	9
May	7:01	18:00	11
June	6:01	19:00	13
July	6:01	19:00	13
August	7:01	18:00	11
September	8:01	17:00	9
October	9:01	16:00	7
November	11:01	14:00	3
December	12:01	13:00	1

Table S4. Day time CO_2 fumigation. Mean ± standard deviations of three months for each ring. Duration times for CO_2 fumigation are in Table S3. E – elevated, A – ambient.

	CO_2 (ppm)					
	A1	A2	A3	E1	E2	E3
7. 2. - 7. 5. 2013	407.84 ± 14.79	407.39 ± 15.42	406.10 ± 15.29	493.17 ± 58.05	436.98 ± 25.67	418.73 ± 21.95
4. 4. - 4. 7. 2013	394.72 ± 16.51	391.62 ± 17.85	393.55 ± 23.65	497.41 ± 65.04	441.51 ± 73.68	441.76 ± 115.38
30. 6. - 31. 9. 2013	395.15 ± 21.46	391.63 ± 25.15	397.86 ± 28.60	484.65 ± 60.74	682.41 ± 162.52	710 .20 ± 165.29

Table S5. Soil moisture (%) of week prior to sampling. Values are mean ± standard deviations of 7 days and 4 replicates per ring. E – elevated, A – ambient.

	Moisture (%)					
	A1	A2	A3	E1	E2	E3
7. 5. 2013	41.58 ± 5.04	46.74 ± 10.24	41.73 ± 6.37	41.68 ± 3.74	46.93 ± 7.03	39.37 ± 2.76
8. 5. 2013	41.43 ± 4.59	46.92 ± 10.25	41.68 ± 6.15	41.91 ± 3.63	47.11 ± 6.70	39.73 ± 2.60
4. 7. 2013	35.08 ± 3.87	45.85 ± 8.19	35.69 ± 6.18	37.37 ± 5.60	42.63 ± 4.20	37.70 ± 5.32
30. 9. 2013	37.50 ± 3.23	34.64 ± 4.49	32.78 ± 3.93	37.53 ± 4.78	41.77 ± 5.42	38.34 ± 3.46

Table S6 (uploaded as a separate file). Results of Wald tests on multivariate models relating OTU abundances to CO_2 treatment. CO_2 coefficients are relative to the ambient treatment, meaning that a positive coefficient indicates increased abundance under elevated CO_2 and a negative coefficient, a decreased abundance under elevated CO_2.

Table S7 (uploaded as a separate file). Coefficients from multivariate GLMs and *P*-values from Wald tests relating AM fungal family abundances to CO_2 treatment. Models treated ambient samples as the reference level, meaning a positive coefficient for elevated CO_2 would indicate increased abundance relative to ambient samples.

Table S8. Negative binomial generalised linear mixed model, with sampling time*CO_2 interaction term, and block specific random intercept. May and ambient samples used as reference levels.

Variable	Estimated coefficient	Standard error	*Z*-statistic	*P*-value
September	-0.09	0.13	-0.67	0.50
July	0.05	0.13	0.38	0.70
eCO_2	0.02	0.12	0.13	0.89
September : eCO_2	-0.06	0.18	-0.31	0.76
July : eCO_2	0.02	0.18	0.09	0.93

Table S9 (uploaded as a separate file). Coefficients from multivariate GLM models and *P*- values calculated by Wald tests on AM fungal OTU abundances in relation to CO_2 treatment and sampling date. CO_2 coefficients are relative to ambient and May samples, meaning a positive coefficient indicates an increase in mean abundance in elevated CO_2 conditions or in other months.

Table S10 (uploaded as a separate file). Coefficients from multivariate GLMs and *P*-values from Wald tests relating AM fungal family abundances to CO_2 treatment and sampling date. As before, May and ambient samples served as reference levels in the models. Therefore, a positive coefficient for September or elevated CO_2 would indicate increased abundance relative to May or ambient samples, respectively.

References

Jäger HJ, Schmidt SW, Kammann C, Grünhage L, Müller C, Hanewald K (2003) The University of Giessen Free-Air Carbon Dioxide Enrichment Study: Description of the Experimental Site and of a New Enrichment System. *Journal of Applied Botany - Angewandte Botanik,* **77**, 117–127.

Trouvelot A, Kough JL, Gianinazzi-Pearson V (1986) Mesure du taux de mycorhization VA d'un système radiculaire. Recherche de méthodes d'estimation ayant une signification functionnelle. In: *Les Mycorhizes: Physiologie et Génétique*. Gianinazzi-Pearson V, and Gianinazzi S, editors. Paris: INRA Press, pp. 217–221.
